# A strongly fluorescent Ni^II^ complex with 2-(2-hy­droxy­eth­yl)pyridine ligands: synthesis, characterization and theoretical analysis and comparison with a related polymeric Cu^II^ complex

**DOI:** 10.1107/S2056989018009301

**Published:** 2018-07-06

**Authors:** Ouahida Zeghouan, Mohamed AbdEsselem Dems, Seifeddine Sellami, Hocine Merazig, Jean Claude Daran

**Affiliations:** a Unité de Recherche Chimie de l’Environnement et Moléculaire Structurale, ’CHEMS’, Faculté des Sciences Exactes, Campus Chaabet Ersas, Université Frères Mentouri Constantine 1, 25000 Constantine, Algeria; b Centre de Recherche en Biotechnologie, Constantine, Algeria; c Laboratoire de Chimie des Matériaux et des Vivants: Activité, Réactivité, Université Hadj-Lakhdar Batna, Algeria; d Laboratoire Pollution et Traitement des Eaux, Département de Chimie, Faculté des Sciences Exactes, Université Frères Mentouri Constantine 1, 25000 Constantine, Algeria; e Laboratoire de Chimie de Coordination, UPR-CNRS 8241, 205 route de Narbonne, 31077 Toulouse Cedex 4, France

**Keywords:** transition metal, fluorescence, blue-light emission, TDDFT, crystal structure

## Abstract

The synthesis and characterization of di­aqua­bis­[2-(2-hy­droxy­eth­yl)pyridine-κ^2^ *N*,*O*)nickel(II) dinitrate, [Ni(C_7_H_9_NO)_2_(H_2_O)_2_](NO_3_)_2_, under ambient conditions is reported and compared with *catena*-poly[[bis­[2-(2-hy­droxy­eth­yl)pyridine-κ^2^ *N*,*O*]copper(II)]-μ-sulfato-κ^2^ *O*:*O*′], [Cu(C_7_H_9_NO)_2_(SO_4_)]_*n*_ [Zegh­ouan *et al.* (2016 ▸). Private communication (refcode 1481676). CCDC, Cambridge, England]. In the two complexes, the 2-(2-hy­droxy­eth­yl)pyridine ligands coordinate the metal ions through the N atom of the pyridine ring and the O atom of the hy­droxy group, creating a chelate ring. The Ni^II^ or Cu^II^ ion lies on an inversion centre and exhibits a slightly distorted *M*O_4_N_2_ octa­hedral coordination geometry, build up by O and N atoms from two 2-(2-hy­droxy­eth­yl)pyridine ligands and two water mol­ecules or two O atoms belonging to sulfate anions. The sulfate anion bridges the Cu^II^ ions, forming a polymeric chain. The photoluminescence properties of these complexes have been studied on as-synthesized samples and reveal that both compounds display a strong blue-light emission with maxima around 497 nm. From DFT/TDDFT studies, the blue emission appears to be derived from the ligand-to-metal charge-transfer (LMCT) excited state. In addition, the IR spectroscopic properties and thermogravimetric behaviours of both complexes have been investigated.

## Abstract 2

A novel mononuclear nickel complex coordinated by 2-(2-hy­droxy­eth­yl)pyridine has been synthesized and structurally characterized by X-ray diffraction techniques and photoluminescence spectroscopy. TDDFT calculations have been performed to rationalize the structure explored for this and a related polymeric Cu complex.

## Chemical context   

A wide variety of nitro­gen-containing heterocyclic ligands has been used to construct coordination complexes (Lin *et al.*, 2015 ▸; Kim *et al.*, 2015 ▸; Huang *et al.*, 2015 ▸). In particular, pyridine alcohol derivatives and their metal complexes have been studied extensively in recent years, focusing on the rational design and synthesis of coordination monomers and polymers because of their intriguing structural features as well as potential applications in catalysis and fluorescence and as chemical sensors (Ley *et al.*, 2010 ▸). Moreover, luminescent compounds have also attracted attention because of their applications, particularly in modern electronics, as materials for producing organic light-emitting diodes (OLEDs) (Kelley *et al.*, 2004 ▸). The 2-(2-hy­droxy­eth­yl)pyridine (hep-H) ligand may adopt many coordinating variants because of its donating capabilities: *N*-monodentate (*N*) (Martínez *et al.*, 2007 ▸), *N*,*O*-chelating (^2^ *N*,*O*) (Antonioli *et al.*, 2007 ▸); deprotonated chelating (^2^ *N*,*O*) (Antonioli *et al.*, 2007 ▸) and bridging (*N*:*O*) (Antonioli *et al.*, 2007 ▸), ^2^ *N*,*O*:*O* ^7^and ^2^ *N*,*O*:*O*:*O* (Wang *et al.*, 2010 ▸) or simultaneously ^2^ *N*,*O*:*O* and ^2^ *N*,*O*:*O*:*O* bridging (Stamatatos, Boudalis *et al.*, 2007 ▸).
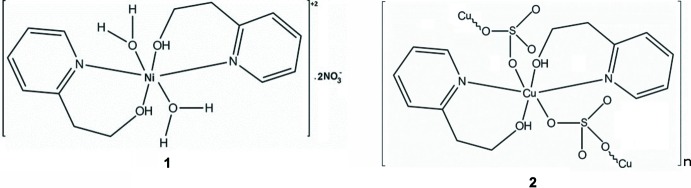


We are in particular inter­ested in the hep-H ligand, which has attracted much attention in biology and chemistry because it is a useful model and for its practical applications (Kong *et al.*, 2009 ▸; Mobin *et al.*, 2010 ▸). The hep-H ligand could be a good candidate to construct simultaneously nitro­gen heteroaromatic alcohol coordination monomers and polymers with inter­esting magnetic behaviour. On the other hand, with Ni^II^ and Cu^II^ metals, the hep-H ligand could also be a desirable candidate for fluorescent materials. The flexible coordination sphere around the Ni^II^ and Cu^II^ ions, in combination with steric and packing forces, is one of the effects that gives rise to a wide structural diversity in Ni^II^/Cu^II^ coordination chemistry (Comba & Remenyi, 2003 ▸).

The combination of multidentate ligands with suitable cations has led to a large number of novel mononuclear and polynuclear complexes. In this study, by reacting the flexible hep-H ligand with Ni(NO_3_)_2_·6H_2_O, we have successfully obtained the monomeric Ni^II^ complex di­aqua­bis­[2-(2-hy­droxy­eth­yl)pyridine-κ^2^ *N*,*O*)nickel(II) dinitrate, [Ni(C_7_H_8_NO)_2_(H_2_O)_2_](NO_3_)_2_ (**1**). The related polymeric complex, *catena*-poly[[bis­[2-(2-hy­droxy­eth­yl)pyridine-κ^2^ *N*,*O*]copper(II)]-μ-sulfato-κ^2^ *O*:*O*′], [Cu(C_7_H_8_NO)_2_(SO_4_)]_*n*_ (Zeghouan *et al.*, 2016 ▸; Zienkiewicz-Machnik *et al.*, 2016 ▸) had previously been obtained by reacting the hep-H ligand with Cu(SO_4_)_2_·6H_2_O. Herein we compare their structures, IR spectra, thermostability, fluorescence and absorption properties and tge results of a theoretical study performed using TDDFT calculations.

## Structural commentary   

**Figure 1:**
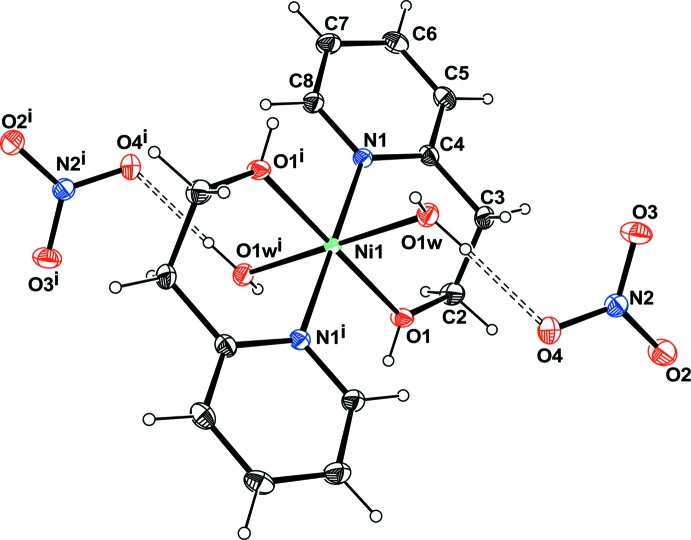
View of the Ni complex, with the atom-numbering scheme. Displacement ellipsoids are drawn at the 50% probability level and H atoms are shown as circles of arbitrary radii. Hydrogen bonds are shown as dashed lines. [Symmetry code: (i): − *x* + 1, −*y* + 1, −*z* + 2].

In the mononuclear title Ni^II^ complex **1** as well as in the polymeric Cu^II^ complex **2** (Zeghouan *et al.*, 2016 ▸; Zienkiewicz-Machnik *et al.*, 2016 ▸), the metal ions are located on inversion centers with the neutral hep-H mol­ecule acting as a bidentate ligand in a ^2^ *N*,*O* fashion and forming the equatorial plane of an octa­hedron, the apex of which is occupied by the water mol­ecules in the case of the Ni^II^ complex or an O atom of an SO_4_^2−^ anion in the Cu^II^ complex (Figs. 1 ▸ and 2 ▸). The main difference between the two structures is the occurrence of the SO_4_^2−^ anion in **2**, which links complex mol­ecules, forming a polymeric chain. Moreover, in this structure the asymmetric unit contains two half mol­ecules of the complex. In the Ni^II^ complex, two nitrate anions balance the charges. The coordination environment around the nickel ions can be described as a nearly perfect octa­hedron. The O1—Ni1—N1 [88.68 (3)°], N1—Ni1—O1*W* [90.00 (4)°] and O1—Ni1—O1*W* [89.26 (4)°] angles are all very close to 90° (Table 1 ▸). The two hep-H ligands are *trans* with respect to each other. The hydroxyl O atom and the pyridine N atoms define the equatorial plane while the water mol­ecules occupy the apices. In the case of the Cu^II^ complex, the octa­hedron is slightly distorted with the angles around the metal ranging from 83.39 (5) to 96.62 (5)°. This distortion might result from the influence of the SO_4_^2−^ linking the Cu complex to form a polymeric chain.

In both complexes, the chelate ring displays a twist-boat conformation with puckering parameters θ = 81.9° and φ = 162° for **1** and θ = 79.2° and φ = 159.9° and θ = 87.75° and φ= 176.08° for the two mol­ecules of **2**.

## Supra­molecular features   

**Figure 3:**
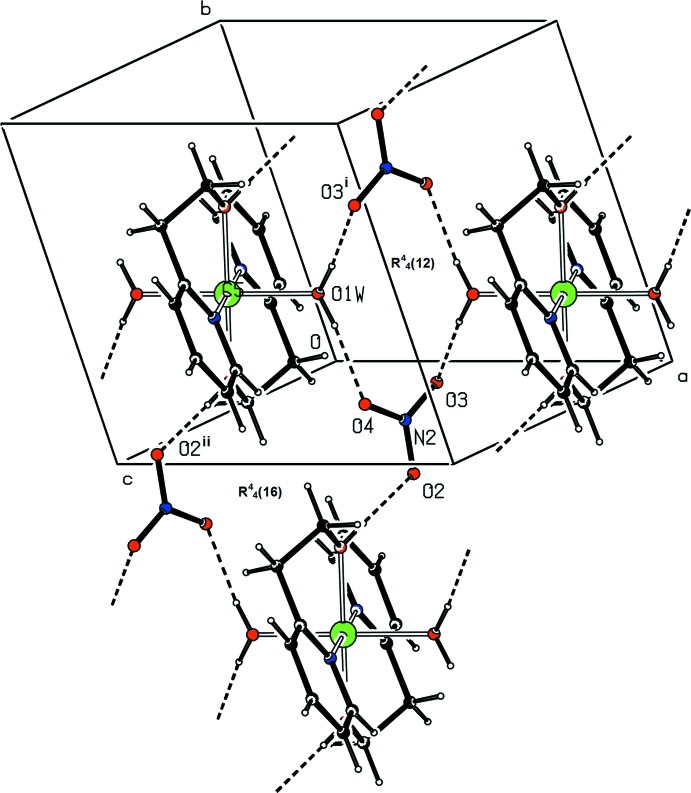
Partial view of the packing in the Ni^II^ complex showing the O—H⋯O hydrogen bonds (dashed lines) and the formation of the 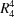
(12) and 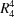
(16) graph-set motifs. [Symmetry codes: (i) − *x* + 2, −*y* + 1, −*z* + 2; (ii) −*x* + 1, −*y*, −*z* + 2].

Although not coordinated to the Ni atom, the nitrate anion in **1** participates in the packing motif. The hydroxyl group and water mol­ecules are involved in strong O—H⋯O hydrogen bonds (Table 2 ▸) with the O atoms of the nitrate anions, resulting in the formation of 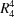
(12) and 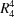
(16) graph-set motifs, as shown in Fig. 3 ▸, building up a three-dimensional network. C—H⋯O hydrogen bonds also occur.

## Database survey   

A search of the Cambridge Structural Database (CSD, Version 5.36; Groom *et al.*, 2016 ▸) based on an Ni(hep-H)_2_O_2_ fragment gave 11 hits for closely related structures with an octa­hedral Ni complex, located on an inversion center, coordinated by two chelating *N*,*O* hep-H ligands in the equatorial plane and two O atoms of different ligands at the apices. A comparison of the Ni—N and Ni—O bond lengths as well as of the dihedral angles between the equatorial NiO_2_N_2_ plane and the pyridine ring is displayed in Table 3 ▸. There are no notable differences between the Ni—O(H) distances, which range from 2.057 (2) to 2.114 (1) Å, and the Ni—O(ligand) bonds, ranging from 2.052 (1) to 2.112 (2) Å. Clearly the organic substituent attached to the O atom in the axial position has no real influence on the Ni—O(*R*) bond length. The dihedral angles between the pryridine ring and the NiN_2_O_2_ basal square plane range from 28.3 to 37.6°. The largest angle is observed for two polymeric structures in which the succinato or adipato organic ligand bridge the Ni atoms, forming a chain; this is possibly related to steric effects. A similar search on the Cu(Hep-H)_2_O_2_ fragment gave seven hits. The major difference observed with the related Ni complexes is the large discrepancy in the Cu—O(H) bond lengths, which range from 2.012 (2) to 2.428 (2) Å and the Cu—O(*R*) lengths, ranging from 1.982 (1) to 2.387 (4) Å. The difference observed between the Cu—O(H) and Cu—O(*R*) bond lengths might be due to the Jahn–Teller effect. The dihedral angles between the pryridine ring and the CuN_2_O_2_ basal square plane, ranging from 26 to 38°, are close to those found in the Ni complexes. Similar twist-boat conformations are observed in all of the related Ni and Cu complexes bearing the hep-H ligand (Table 3 ▸).

## Thermogravimetric and differential thermal analysis   

Thermal analyses were performed on a SETARM 92-16.18 PC/PG 1 instrument from 303 to 1273 K under a dynamic air atmosphere and under nitro­gen at 200.0 ml min^−1^ with a heating rate of 283 K min ^−1^. The stability of the two complexes was measured by TGA and the experimental results are in agreement with the calculated data.

**Figure 4:**
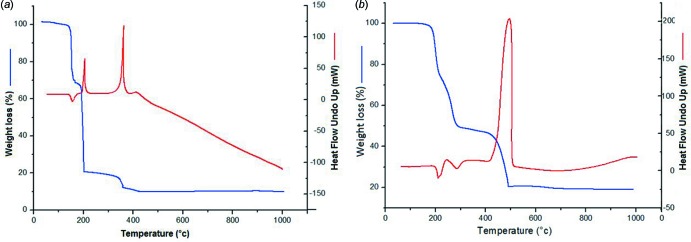
The thermogravimetric (TG) and differential thermal analysis (DTA) curves for (*a*) the monomer and (*b*) the polymer.

The TG curve for **1** (Fig. 4 ▸ *a*) shows that the monomer is stable up to 424 K with the first weight loss of 33.55% (calculated 34.21%) at 303 −438 K corresponding to the loss of two coordinated water mol­ecules and the organic hep-H ligand. The second loss of 47.51% (calculated 40.02%) at 438–488 K corresponds to the loss of the second hep-H ligand and the nitrate anion, and then the second nitrate anion decomposes (DP/P = 12.14%, calculated =13.34%). In addition, the corresponding endothermic and exothermic peaks (at 424.26, 475.06 and 631.85 K) in the differential scanning ATD curve also record the processes of weight loss. As illustrated in Fig. 4 ▸ *b*, the TG curve for **2** shows that the polymer is stable up to 470 K with the first weight loss of 24.16% (calculated 23.66%) at 470–483 K corresponding to the loss of the sulfate anion and the second loss of 29.42% (calculated 30.30%) at 483–573 K to the loss of the first hep-H ligand, and then the second hep-H ligand decomposes (DP/P = 28.55%, calculated =30.30%). In addition, the corresponding endothermic and exothermic peaks (at 473, 558 and 773 K) in the differential scanning ATD curve also record the processes of weight loss.

## Luminescence properties   

**Figure 5:**
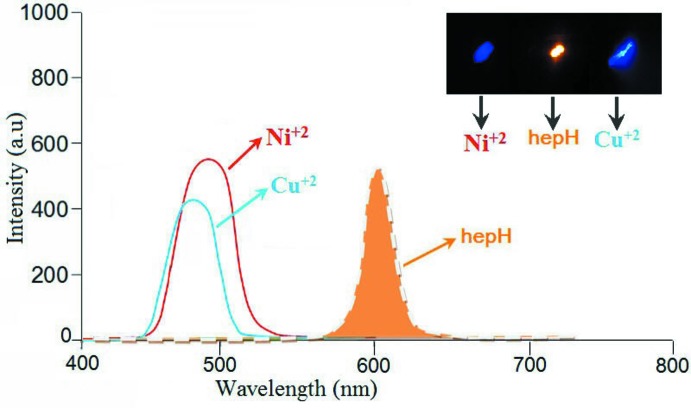
The fluorescence spectrum of the hep-H ligand and the title compounds (excitation at 250 and 269.70 nm for the complexes and hep-H, respectively)

Photoluminescence spectra were measured using a Cary Eclipse (Agilent Technologies) fluorescence spectrophotometer with quartz cell (1 × 1 cm^2^ cross-section) equipped with a xenon lamp and a dual monochromator. The measurements were carried out at ambient temperature (298 K) with the slit_ex/em_ = 10 nm/10 nm. The photoluminescence properties of **1**, **2** and free hep-H in an ethanol–water (*v*/*v* = 1:1) solution were investigated in the visible region. As shown in Fig. 5 ▸, free hep-H displays orange emission with a band at 496.06 nm (excited at 269.70 nm), which may be assigned to a π–π* electronic transition. When hep-H is combined with Ni^II^ or Cu^II^ in **1** or **2**, an intense blue emission band is seen at λ_em_/λ_ex_ = 498.03 nm/250.93 nm or 496.96 nm/250.00 nm respectively. This should probably be assigned to the π–π* charge-transfer inter­action of the hep-H ligands. The observed blue shift of the emission maximum between **1**, **2** and free hep-H is considered to originate mainly from the influence of the coordination of the metal atoms to the hep-H ligand (Leitl *et al.*, 2016 ▸). Thus, these compounds may be candidates for blue-light luminescent materials which suggests that more transition metal, pyridine alcohol compounds with good luminescent properties can be developed.

## TDDFT calculations   

In an effort to better understand the nature of the electronic transitions exhibited by compounds **1** and **2**, DFT calculations using the Amsterdam density function (ADF) software (Baerends *et al.*, 1973 ▸) along with generalized gradient approximations, exchange and correlation functional GGA (PBE) (Perdew *et al.*, 1997 ▸), employing the TZP (triple zeta polarized) basis set. The singlet excited state was optimized using time-dependent density functional theory calculations (TDDFT) (Bauernschmitt & Ahlrichs, 1996 ▸; Gross & Kohn, 1990 ▸; Gross *et al.*, 1996 ▸).

**Figure 6:**
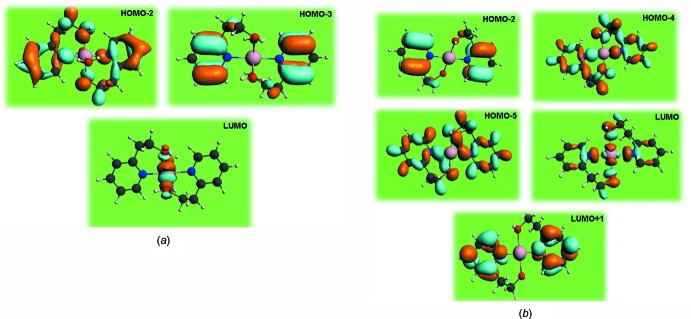
Plots of the mol­ecular orbitals dominating the contribution of the low-energy transitions for (*a*) the monomer and (*b*) the polymer.

The ground-state geometry of **1** and **2** was adapted from the X-ray data. The calculated structural parameters show a good agreement with the original X-ray diffraction data (Table 1 ▸); the root-mean-square deviation *f* between the X-ray and the DFT structure for non-hydrogen atoms is 0.603 and 0.620 Å for **1** and **2**, respectively. The computed absorption bands, dominant transitions, characters, and oscillator strengths (*f*) are given in Table 4 ▸. As shown in this table, two absorption features are predicted for the monomer; these mainly consist of absorption peaks located at λ = 286 and 280 nm, resulting from the HOMO-2 to LUMO transition and the HOMO-3 to LUMO transition, which is attributed to a ligand–metal charge transfer (LMTC) (Fig. 6 ▸ *a*). Three absorption features are predicted in the polymer, consisting mainly of absorption peaks that are located at λ = 507, 443 and 244 nm, resulting from HOMO-4 to LUMO, HOMO-5 to LUMO and HOMO-2 to LUMO transitions, which are attributed to a ligand–metal charge transfer (LMTC) (Fig. 6 ▸ *b*). The HOMO–LUMO energy gap was found to be 4.33, 4.42 for the transitions in **1** and 2.44, 4.03, 5.81 ev for the transitions in **2**.

## Synthesis and crystallization   

All chemicals and solvents were commercially purchased and used as received. The infrared spectra were recorded on a Perkin–Elmer spectrometer at room temperature in the range of 4000–500 cm^−1^.

The hep-H ligand was obtained from commercial sources. The synthesis of the two compounds followed the same procedures as previously described for the Co^II^ analog (Zeghouan *et al.*, 2013 ▸). (2-Hy­droxy­eth­yl)pyridine (10.0 mmol, 1.67 g) was reacted in a mixture of ethanol–water (*v*/*v* = 1:1) with Ni(NO_3_)_2_·6H_2_O (10.0 mmol, 2.50 g) for the Ni^II^ analogue and with Cu(SO_4_)_2_·6H_2_O (10.0 mmol, 2.3 g) for the Cu^II^ analogue. The solutions were maintained under agitation for 24 h at room temperature. Green prisms of the monomer and green prisms of the polymer were obtained by slow evaporation of the solvents within three weeks. The crystals formed were filtered and washed with 15 ml of water.

**IR** (cm^−1^, pure crystals of compounds without KBr): Ni analogue: 3389 (*vs*), 3124 (*vs*), 2862 (*m*), 2764 (*m*), 2360 (*m*), 1658 (*m*), 1442 (*m*), 1371 (*vs*), 1306 (*vs*), 1084 (*m*), 1021 (*m*), 763 (*m*), 586 (*m*). Cu analogue: 3392 (*vs*), 3127 (*s*), 2911 (*m*), 1655 (*m*), 1609 (*m*), 1572 (*w*), 1493 (*w*), 1444 (*w*), 1373 (*vs*), 1356 (*vs*), 1313 (*m*), 1159 (*m*), 1082 (*m*), 1023 (*m*), 907 (*w*), 860 (*s*), 764 (*m*), 706 (*s*), 643 (*s*).

## Refinement   

Crystal data, data collection and structure refinement details are summarized in Table 5 ▸. O-bound H atoms were located in a difference-Fourier map and refined with O—H restrained to 0.85 (1) Å, with *U*_i__so_(H) = 1.5*U*_eq_(O). For the water mol­ecule a further H⋯H distance restraint of 1.39 (2) Å was used. C-bound H atoms were placed at calculated positions with C—H = 0.93 Å (aromatic H atoms) and 0.97 Å (methyl­ene H atoms), and refined in riding mode with *U*_iso_(H) = 1.2*U*_eq_(C). Four reflections were omitted from the refinement.

## Supplementary Material

Crystal structure: contains datablock(s) global, I. DOI: 10.1107/S2056989018009301/vn2135sup1.cif

Structure factors: contains datablock(s) I. DOI: 10.1107/S2056989018009301/vn2135Isup3.hkl

CCDC reference: 1481640

Additional supporting information:  crystallographic information; 3D view; checkCIF report

## Figures and Tables

**Figure 2:**
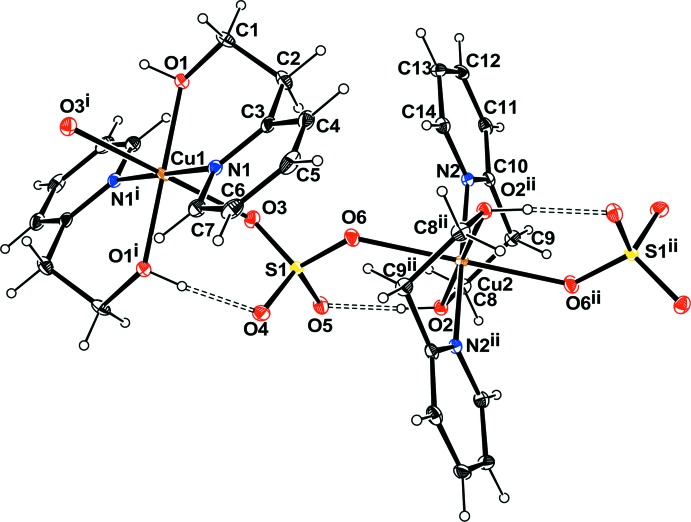
Partial view of the polymer chain in the Cu compound, with displacement ellipsoids drawn at the 50% probability level. Hydrogen bonds are shown as dashed lines. [Symmetry code: (i) −*x* + 1, −*y* + 2, −*z* + 2; (ii) −*x*, −*y* + 1, −*z* + 1].

**Table 1:** Comparison of experimental and calculated distances and angles (Å, °) in **1** and **2**

**1**		
Ni1—O1	2.0622 (14)	2.07
Ni1—O1*W*	2.0831 (15)	2.10
Ni1—N1	2.1019 (14)	2.12
O2—N2	1.2520 (14)	1.23
O3—N2	1.2548 (12)	1.22
O4—N2	1.2537 (12)	1.27
		
O1—Ni1—O1*W*	90.74 (4)	93.00
O1—Ni1—N1	88.68 (3)	87.00
O1—Ni1—O1*W* ^i^	89.26 (4)	91.8
O1—Ni1—N1^i^	91.32 (3)	92.3
O1*W*—Ni1—N1	90.00 (4)	92
O1^i^—Ni1—O1*W*	89.26 (4)	87.00
O1*W*—Ni1—N1^i^	90.00 (4)	91.8
O1^i^—Ni1—N1	91.32 (3)	92.3
O1*W* ^i^—Ni1—N1	90.00 (4)	91.8
O1^i^—Ni1—O1*W* ^i^	90.74 (4)	87.5
		
O1*W*—Ni1—O1—C2	−79.99 (10)	80.02
N1^i^—Ni1—O1—C2	−170.01 (10)	166.70
O1—Ni1—N1—C4	−29.33 (10)	31.30
O1—Ni1—N1—C8	151.96 (9)	−147.60
O1*W* ^i^—Ni1—N1—C4	−118.59 (9)	114.9
O1*W* ^i^—Ni1—N1—C8	62.70 (9)	−64.00
		
**2**		
Cu1—O1	2.01	2.08
Cu1—N1	2.02	1.99
Cu1—O1^i^	2.01	2.08
Cu1—N1^i^	2.02	1.99
Cu2—O2	2.05	2.08
		
N2—Cu2—N2^ii^	180	180.00
O1—Cu1—N1	92.39	90.60
O1—Cu1—O1^i^	180	180.00
		
O3—Cu1—O1—C1	−93.9	−98
O1—Cu1—N1—C7	−151.03	−153
N2—Cu2—O2—C8	151.24	148
Cu1—O1—C1—C2	−37.55.	−40
C7—N1—C3—C2	177	179
C7—N1—C3—C4	−0.5	−0.4

**Table 2:** Hydrogen-bond geometry (Å, °)

*D*—H⋯*A*	*D*—H	H⋯*A*	*D*⋯*A*	*D*—H⋯*A*
O1*W*—H2*W*⋯O3^i^	0.84	1.95	2.7870 (16)	176
O1*W*—H2*W*⋯N2^i^	0.84	2.68	3.455 (2)	153
O1*W*—H1*W*⋯O4	0.85	1.93	2.7720 (19)	174
O1—H1⋯O2^ii^	0.82	1.88	2.6952 (15)	172
O1—H1⋯N2^ii^	0.82	2.65	3.4208 (17)	159
C2—H2*B*⋯O3^iii^	0.97	2.64	3.378 (2)	133
C3—H3*A*⋯O1*W*	0.97	2.55	3.2278 (17)	127
C8—H8⋯O1^iv^	0.93	2.49	3.0136 (19)	116
C8—H8⋯O4^iv^	0.93	2.66	3.4448 (18)	143
C5—H5⋯O2^v^	0.93	2.41	3.3076 (19)	163

Symmetry codes: (i) 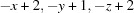; (ii) 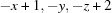; (iii) 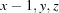; (iv) 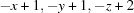; (v) 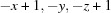.

**Table 3:** Comparison of selected geometrical parameters (%, Å, °) for Ni^II^ and Cu^II^ complexes bearing the hep-H ligand Δ is the dihedral angle between the basal *M*O_2_N_2_ square plane and the pyridine ring.

Ref.	*R*-factor	*M*—N	*M*—OH	*M*—O(*R*)	Δ	θ	φ
**1**	1.80	2.102 (1)	2.062 (1)	2.083 (2)	28.28 (4)	81.9 (1)	162.6 (1)
BOZJAD^*a*^	3.80	2.102 (2)	2.065 (3)	2.084 (3)	28.4 (1)	80.6 (3)	163.4 (3)
HULYAO^*b*^	3.22	2.073 (1)	2.064 (1)	2.085 (1)	30.37 (6)	78.7 (1)	156.3 (1)
EJEZE*Z* ^*c*^	2.58	2.082 (1)	2.089 (1)	2.090 (1)	30.88 (6)	99.0 (1)	346.8 (1)
FEFWIY^*d*^	3.13	2.100 (2)	2.088 (1)	2.072 (2)	30.4 (1)	96.8 (2)	349.7 (2)
FEFWIY01^*d*^	3.05	2.090 (1)	2.104 (1)	2.064 (1)	31.86 (8)	95.9 (1)	354.6 (2)
FEFWIY02^*d*^	2.59	2.096 (1)	2.085 (1)	2.064 (1)	30.51 (7)	97.9 (1)	346.1 (1)
BOZJO*R* ^*a*^	3.75	2.078 (2)	2.096 (1)	2.063 (2)	37.6 (1)	89.2 (2)	175.3 (2)
BOZJU*X* ^*a*^	3.03	2.083 (1)	2.114 (1)	2.052 (1)	35.43 (8)	94.3 (1)	352.5 (2)
BOZKAE^*a*^	4.36	2.098 (2)	2.096 (2)	2.064 (2)	29.9 (1)	81.9 (2)	160.4 (2)
RAJQO*L* ^*e*^	4.07	2.083 (2)	2.057 (2)	2.112 (2)	31.9 (1)	84.5 (2)	167.7 (2)
							
**2** NABBEA01^*f*^	1.9	2.025 (2) 1.988 (2)	2.012 (2) 2.055 (1)	2.380 (1) 2.298 (1)	28.5 (1) 38.0 (1)	79.2 (1) 87.8 (1)	159.9 (1) 176.2 (1)
NABBEA^*g*^	5.2	1.993 (4) 2.031 (4)	2.070 (4) 2.016 (4)	2.298 (4) 2.387 (4)	37.5 (2) 28.8 (2)	87.5 (3) 100.3 (4)	175.9 (3) 340.6 (4)
HAYHAS^*h*^	2.8	2.032 (2)	2.422 (1)	1.982 (1)	29.50 (7)	81.9 (1)	171.9 (1)
IRERED^*i*^	4.04	2.017 (2)	2.385 (2)	2.025 (2)	31.0 (1)	94.4 (2)	356.0 (2)
OJOBAQ^*j*^	2.35	2.009 (1)	2.041 (1)	2.312 (1)	33.96 (4)	98.6 (1)	340.0 (1)
SOJGAB^*k*^	3.52	2.029 (2)	2.428 (2)	1.998 (1)	25.97 (8)	101.4 (2)	346.6 (2)
UGAROK^*l*^	3.44	2.021 (2) 2.030 (2)	2.019 (2) 2.024 (2)	2.357 (2) 2.346 (2)	31.4 (1) 32.5 (1)	95.9 (2) 80.6 (2)	345.3 (2) 167.0 (2)

Notes: (*a*) Trdin *et al.* (2015 ▸); (*b*) Hamamci *et al.* (2002 ▸); (*c*) Yilmaz *et al.* (2011 ▸); (*d*) Trdin & Lah (2012 ▸); (*e*) Çolak *et al.* (2017 ▸); (*f*) Zeghouan *et al.* (2016 ▸); (*g*) Zienkiewicz-Machnik *et al.* (2016 ▸); (*h*) Lapanje *et al.* (2012 ▸); (*i*) Pothiraja *et al.* (2011 ▸); (*j*) Yilmaz *et al.* (2003 ▸); (*k*) Caglar *et al.* (2014 ▸); (*l*) Yeşılel *et al.* (2009 ▸).

**Table 4:** The calculated optical transition energies (nm) and their corresponding oscillator strengths (*f*) (ev) for **1** and **2**

λ	*f*	*E*	Transition	Type
**1**				
286	0.03	4.33	HOMO-2 to LUMO	LMTC
280	0.01	4.42	HOMO-3 to LUMO	LMTC
**2**				
507	0.009	2.44	HOMO-4 to LUMO	LMTC
443	0.08	4.03	HOMO-5 to LUMO	LMTC
244	0.07	5.81	HOMO-2 to LUMO+1	LLTC

**Table 5:** Experimental details

Crystal data	
Chemical formula	[Ni(C_7_H_9_NO)_2_(H_2_O)_2_](NO_3_)_2_
*M*_r_	465.05
Crystal system, space group	Triclinic, *P* 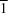
Temperature (K)	293
*a*, *b*, *c* (Å)	7.782 (5), 8.185 (5), 8.811 (5)
α, β, γ (°)	96.785 (5), 113.856 (5), 109.140 (5)
*V* (Å^3^)	464.0 (5)
*Z*	1
Radiation type	Mo *K*α
μ (mm^−1^)	1.11
Crystal size (mm)	0.18 × 0.11 × 0.08

Data collection	
Diffractometer	Bruker APEXII
No. of measured, independent and observed [*I* > 2σ(*I*)] reflections	2478, 2478, 2471
*R*_int_	0.019
(sin θ/λ)_max_ (Å^−1^)	0.685

Refinement	
*R*[*F* ^2^ > 2σ(*F* ^2^)], *wR*(*F* ^2^), *S*	0.018, 0.051, 1.08
No. of reflections	2478
No. of parameters	133
No. of restraints	4
H-atom treatment	H atoms treated by a mixture of independent and constrained refinement
Δρ_max_, Δρ_min_ (e Å^−3^)	0.42, −0.29

Computer programs: *APEX2* and *SAINT* (Bruker, 2006 ▸), *SIR2002* (Burla *et al.*, 2003 ▸), *SHELXL* (Sheldrick, 2015 ▸), *ORTEPIII* (Burnett & Johnson, 1996 ▸), *ORTEP-3 for Windows* and *WinGX* (Farrugia, 2012 ▸), *PLATON* (Spek, 2009 ▸) and *Mercury* (Macrae *et al.*, 2006 ▸).

## supplementary crystallographic information

### Crystal data

**Table:**

[Ni(C_7_H_9_NO)_2_(H_2_O)_2_](NO_3_)_2_	*Z* = 1
*M**_r_* = 465.05	*F*(000) = 242
Triclinic, *P*1	*D*_x_ = 1.664 Mg m^−^^3^
Hall symbol: -P 1	Mo *K*α radiation, λ = 0.71073 Å
*a* = 7.782 (5) Å	Cell parameters from 1536 reflections
*b* = 8.185 (5) Å	θ = 2.8–33.8°
*c* = 8.811 (5) Å	µ = 1.11 mm^−^^1^
α = 96.785 (5)°	*T* = 293 K
β = 113.856 (5)°	Prism, green
γ = 109.140 (5)°	0.18 × 0.11 × 0.08 mm
*V* = 464.0 (5) Å^3^	

### Data collection

**Table:**

Bruker APEXII diffractometer	2471 reflections with *I* > 2σ(*I*)
Radiation source: fine-focus sealed tube	*R*_int_ = 0.019
Graphite monochromator	θ_max_ = 29.1°, θ_min_ = 3.0°
φ scans	*h* = −11→11
2478 measured reflections	*k* = −11→11
2478 independent reflections	*l* = −12→11

### Refinement

**Table:**

Refinement on *F*^2^	4 restraints
Least-squares matrix: full	Hydrogen site location: mixed
*R*[*F*^2^ > 2σ(*F*^2^)] = 0.018	H atoms treated by a mixture of independent and constrained refinement
*wR*(*F*^2^) = 0.051	*w* = 1/[σ^2^(*F*_o_^2^) + (0.0235*P*)^2^ + 0.1863*P*] where *P* = (*F*_o_^2^ + 2*F*_c_^2^)/3
*S* = 1.08	(Δ/σ)_max_ < 0.001
2478 reflections	Δρ_max_ = 0.42 e Å^−^^3^
133 parameters	Δρ_min_ = −0.29 e Å^−^^3^

### Special details

**Table:**

Geometry. All esds (except the esd in the dihedral angle between two l.s. planes) are estimated using the full covariance matrix. The cell esds are taken into account individually in the estimation of esds in distances, angles and torsion angles; correlations between esds in cell parameters are only used when they are defined by crystal symmetry. An approximate (isotropic) treatment of cell esds is used for estimating esds involving l.s. planes.

### Fractional atomic coordinates and isotropic or equivalent isotropic displacement parameters (Å^2^)

**Table:**

	*x*	*y*	*z*	*U*_iso_*/*U*_eq_	
Ni1	0.500000	0.500000	1.000000	0.01030 (6)	
O1W	0.76119 (11)	0.49746 (9)	0.98827 (9)	0.01538 (14)	
H2W	0.870471	0.591206	1.051410	0.023*	
H1W	0.786623	0.405991	1.002286	0.023*	
O1	0.36368 (11)	0.22139 (9)	0.92461 (9)	0.01563 (14)	
H1	0.312496	0.167495	0.978813	0.023*	
O3	0.88841 (12)	0.18215 (11)	0.80898 (10)	0.02212 (16)	
O4	0.81394 (14)	0.18243 (11)	1.02219 (11)	0.02427 (17)	
O2	0.77192 (14)	−0.06798 (10)	0.86796 (11)	0.02611 (18)	
N1	0.36503 (12)	0.49127 (11)	0.73709 (10)	0.01265 (15)	
N2	0.82483 (13)	0.09922 (11)	0.89982 (11)	0.01520 (16)	
C2	0.26126 (17)	0.10590 (13)	0.75001 (13)	0.01930 (19)	
H2B	0.116946	0.085298	0.694728	0.023*	
H2A	0.268633	−0.009987	0.751370	0.023*	
C3	0.36373 (16)	0.19383 (13)	0.64843 (13)	0.01691 (18)	
H3A	0.511649	0.232402	0.715215	0.020*	
H3B	0.315995	0.104372	0.541211	0.020*	
C7	0.24432 (16)	0.64650 (14)	0.52920 (13)	0.01830 (19)	
H7	0.220254	0.747349	0.506920	0.022*	
C6	0.20270 (16)	0.50690 (15)	0.39547 (13)	0.01914 (19)	
H6	0.151505	0.513073	0.281924	0.023*	
C8	0.32254 (15)	0.63218 (13)	0.69660 (12)	0.01527 (17)	
H8	0.346937	0.724379	0.785476	0.018*	
C4	0.32151 (14)	0.35408 (13)	0.60574 (12)	0.01359 (17)	
C5	0.23876 (16)	0.35837 (14)	0.43420 (12)	0.01737 (18)	
H5	0.207865	0.261841	0.346023	0.021*	

### Atomic displacement parameters (Å^2^)

**Table:**

	*U*^11^	*U*^22^	*U*^33^	*U*^12^	*U*^13^	*U*^23^
Ni1	0.01338 (8)	0.00887 (8)	0.00873 (8)	0.00453 (6)	0.00534 (6)	0.00257 (6)
O1W	0.0160 (3)	0.0134 (3)	0.0175 (3)	0.0064 (3)	0.0085 (3)	0.0041 (3)
O1	0.0221 (3)	0.0099 (3)	0.0129 (3)	0.0034 (3)	0.0092 (3)	0.0027 (2)
O3	0.0254 (4)	0.0203 (4)	0.0214 (4)	0.0055 (3)	0.0140 (3)	0.0093 (3)
O4	0.0394 (5)	0.0201 (4)	0.0242 (4)	0.0169 (3)	0.0210 (4)	0.0073 (3)
O2	0.0401 (5)	0.0120 (3)	0.0290 (4)	0.0060 (3)	0.0232 (4)	0.0038 (3)
N1	0.0150 (3)	0.0124 (3)	0.0109 (3)	0.0058 (3)	0.0062 (3)	0.0035 (3)
N2	0.0148 (4)	0.0143 (4)	0.0157 (4)	0.0055 (3)	0.0069 (3)	0.0042 (3)
C2	0.0266 (5)	0.0106 (4)	0.0149 (4)	0.0034 (4)	0.0088 (4)	0.0006 (3)
C3	0.0241 (5)	0.0144 (4)	0.0140 (4)	0.0097 (4)	0.0097 (4)	0.0026 (3)
C7	0.0214 (5)	0.0195 (5)	0.0160 (4)	0.0103 (4)	0.0081 (4)	0.0090 (4)
C6	0.0200 (4)	0.0248 (5)	0.0116 (4)	0.0086 (4)	0.0065 (4)	0.0072 (4)
C8	0.0186 (4)	0.0144 (4)	0.0136 (4)	0.0076 (3)	0.0075 (3)	0.0046 (3)
C4	0.0144 (4)	0.0139 (4)	0.0121 (4)	0.0049 (3)	0.0069 (3)	0.0026 (3)
C5	0.0188 (4)	0.0198 (4)	0.0111 (4)	0.0065 (4)	0.0067 (3)	0.0018 (3)

### Geometric parameters (Å, º)

**Table:**

Ni1—O1^i^	2.0622 (14)	C2—C3	1.5193 (15)
Ni1—O1	2.0622 (14)	C2—H2B	0.9700
Ni1—O1W^i^	2.0831 (15)	C2—H2A	0.9700
Ni1—O1W	2.0831 (15)	C3—C4	1.5063 (15)
Ni1—N1	2.1019 (14)	C3—H3A	0.9700
Ni1—N1^i^	2.1019 (14)	C3—H3B	0.9700
O1W—H2W	0.8426	C7—C8	1.3861 (15)
O1W—H1W	0.8451	C7—C6	1.3891 (16)
O1—C2	1.4374 (14)	C7—H7	0.9300
O1—H1	0.8184	C6—C5	1.3848 (16)
O3—N2	1.2548 (12)	C6—H6	0.9300
O4—N2	1.2537 (12)	C8—H8	0.9300
O2—N2	1.2520 (14)	C4—C5	1.3930 (15)
N1—C8	1.3485 (14)	C5—H5	0.9300
N1—C4	1.3570 (13)		
			
O1^i^—Ni1—O1	180.0	O1—C2—C3	109.66 (9)
O1^i^—Ni1—O1W^i^	90.74 (4)	O1—C2—H2B	109.7
O1—Ni1—O1W^i^	89.26 (4)	C3—C2—H2B	109.7
O1^i^—Ni1—O1W	89.26 (4)	O1—C2—H2A	109.7
O1—Ni1—O1W	90.74 (4)	C3—C2—H2A	109.7
O1W^i^—Ni1—O1W	180.0	H2B—C2—H2A	108.2
O1^i^—Ni1—N1	91.32 (3)	C4—C3—C2	113.88 (9)
O1—Ni1—N1	88.68 (3)	C4—C3—H3A	108.8
O1W^i^—Ni1—N1	90.00 (4)	C2—C3—H3A	108.8
O1W—Ni1—N1	90.00 (4)	C4—C3—H3B	108.8
O1^i^—Ni1—N1^i^	88.68 (3)	C2—C3—H3B	108.8
O1—Ni1—N1^i^	91.32 (3)	H3A—C3—H3B	107.7
O1W^i^—Ni1—N1^i^	90.00 (4)	C8—C7—C6	118.53 (10)
O1W—Ni1—N1^i^	90.00 (4)	C8—C7—H7	120.7
N1—Ni1—N1^i^	180.0	C6—C7—H7	120.7
Ni1—O1W—H2W	114.9	C5—C6—C7	118.83 (10)
Ni1—O1W—H1W	117.1	C5—C6—H6	120.6
H2W—O1W—H1W	108.6	C7—C6—H6	120.6
C2—O1—Ni1	125.84 (6)	N1—C8—C7	123.30 (9)
C2—O1—H1	107.8	N1—C8—H8	118.3
Ni1—O1—H1	120.3	C7—C8—H8	118.3
C8—N1—C4	117.93 (9)	N1—C4—C5	121.60 (9)
C8—N1—Ni1	118.03 (6)	N1—C4—C3	118.57 (9)
C4—N1—Ni1	124.03 (7)	C5—C4—C3	119.83 (9)
O2—N2—O4	119.76 (9)	C6—C5—C4	119.75 (9)
O2—N2—O3	119.70 (9)	C6—C5—H5	120.1
O4—N2—O3	120.54 (9)	C4—C5—H5	120.1

Symmetry code: (i) −*x*+1, −*y*+1, −*z*+2.

### Hydrogen-bond geometry (Å, º)

**Table:**

*D*—H···*A*	*D*—H	H···*A*	*D*···*A*	*D*—H···*A*
O1*W*—H2*W*···O3^ii^	0.84	1.95	2.7870 (16)	176
O1*W*—H2*W*···N2^ii^	0.84	2.68	3.455 (2)	153
O1*W*—H1*W*···O4	0.85	1.93	2.7720 (19)	174
O1—H1···O2^iii^	0.82	1.88	2.6952 (15)	172
O1—H1···N2^iii^	0.82	2.65	3.4208 (17)	159
C2—H2*B*···O3^iv^	0.97	2.64	3.378 (2)	133
C3—H3*A*···O1*W*	0.97	2.55	3.2278 (17)	127
C8—H8···O1^i^	0.93	2.49	3.0136 (19)	116
C8—H8···O4^i^	0.93	2.66	3.4448 (18)	143
C5—H5···O2^v^	0.93	2.41	3.3076 (19)	163

Symmetry codes: (i) −*x*+1, −*y*+1, −*z*+2; (ii) −*x*+2, −*y*+1, −*z*+2; (iii) −*x*+1, −*y*, −*z*+2; (iv) *x*−1, *y*, *z*; (v) −*x*+1, −*y*, −*z*+1.
